# Factors associated with the utilization of primary care emergency centers in a Spanish region with high population dispersion: a mixed-methods study

**DOI:** 10.1186/1472-6963-14-368

**Published:** 2014-09-03

**Authors:** Belén Sanz-Barbero, Laura Otero-García, Teresa Blasco-Hernández, Miguel San Sebastián

**Affiliations:** National School of Public Health, Health Institute Carlos III, Madrid, Spain; CIBER of Epidemiology and Public Health, CIBERESP, Madrid, Spain; National Centre of Tropical Medicine, Health Institute Carlos III, Madrid, Spain; Department of Public Health and Clinical Medicine Umeå University, Umeå International School of Public Health, Umeå, Sweden

**Keywords:** Primary care emergency center, Health services utilization, Mixed-methods approach, Spain

## Abstract

**Background:**

Adequate access to primary care emergency centers is particularly important in rural areas isolated from urban centers. However, variability in utilization of emergency services located in primary care centers among inhabitants of nearby geographical areas is understudied. The objectives of this study are twofold: 1) to analyze the association between the availability of municipal emergency care centers and utilization of primary care emergency centers (PCEC), in a Spanish region with high population dispersion; and 2) to determine healthcare providers’ perceptions regarding PCEC utilization.

**Methods:**

A mixed-methods study was conducted. Quantitative phase: multilevel logistic regression modeling using merged data from the 2003 Regional Health Survey of Castile and Leon and the 2001 census data (Spain). Qualitative phase:14 in-depth- interviews of rural-based PCEC providers.

**Results:**

Having PCEC as the only emergency center in the municipality was directly associated with its utilization (p < 0.001). Healthcare providers perceived that distance to hospital increased PCEC utilization, and distance to PCEC decrease its use. PCEC users were considered to be predominantly workers and students with scheduling conflicts with rural primary care opening hours.

**Conclusions:**

The location of emergency care centers is associated with PCEC utilization. Increasing access to primary care by extending hours may be an important step toward optimal PCEC utilization. Further research would determine whether lower PCEC use by certain groups is associated with disparities in access to care.

## Background

In recent years several studies have examined the association between the geographic, economic, and social context of the area of residence and individuals’ health status [1, 2]. Whereas many causes underlie health disparities, unequal access to healthcare services is bound to explain a significant portion of the observed inequality [3]. Nevertheless, the association between the aforementioned context and the individual use of healthcare services is an understudied area of research [4, 5]. As previous research shows, hospital emergency services (HES) utilization is higher in areas of lower socioeconomic level, as well as those with a greater proportion of immigrant population [6, 7]. Regarding the geographic context, as distance from area of residence to the hospital increases, utilization of HES diminishes [8, 9]. However, the variability in the use of primary care emergency centers (PCEC) among nearby health areas is also understudied [10]. Recently published research from Spain shows that variability in utilization of pre-hospital emergency services is substantial. The authors further suggest that such high variability may be related to rurality and the number of PCEC [11]. However, how the availability of emergency centers *(hospital, primary care emergency center)* in geographic areas of high population dispersion is related to PCEC use has not been determined. While adequate PCEC access is particularly important in rural areas isolated from urban centers, inadequate utilization of such centers may lead to higher costs, delayed or ineffective response to emergencies, and loss of continuity of care by general practitioners [12].

The Spanish National Healthcare System is decentralized at the regional level. Primary care services are the entry point to the healthcare system. The National Healthcare System’s goal is to provide universal access to the population independently of socioeconomic status or geographic location [13, 14]. Located in Spain, the region of Castile and Leon is one of the largest European region with the highest population dispersion. The geographical characteristics of the region, described in detail below under “Data and Methods—Quantitative Study”, provide an ideal context in which to study the use of emergency healthcare centers and how it relates to rurality and availability of different healthcare resources.

In summary, the three main objectives of this work are to: 1) explore whether PCEC use varies across municipalities and, if so, what portion of such variability is explained by the availability of emergency care centers in the municipality of residence; 2) examine the association between PCEC use and the availability of emergency care centers (both hospital-based and primary care) in the municipality of residence; and 3) to know the perceptions of the healthcare professionals working at rural PCEC regarding the use of these services and the factors determining such use patterns.

## Methods

To further our understanding of PCEC use, a mixed-methods study was designed [15]. Because the quantitative and qualitative data were collected from different geographical areas, we describe the characteristics of each area under their respective subheadings. First, the quantitative phase of the study consisted of multi-level multivariate analyses of the data constructed from merging three datasets. A regional health survey provided individual-level data, and regional health planning guidelines and the most recent census data provided municipality-level information. Second, to supplement the quantitative data and provide depth to the findings, the qualitative phase of the study consisted of 14 in-depth interviews of healthcare professionals working in different rural PCEC. The concept of rural area uses on that study was based on the Spanish National Institute of Statistic, concretely Census of Population and Housing, that consider rural area all the population entities with less than 2000 inhabitants.

### Quantitative phase of the study

#### Geographical context

The region of Castile and Leon has 2,557,330 inhabitants (27.13 inhabitants/km^2^) distributed across 9 provinces and 2,248 municipalities [16]. Almost 98% of these municipalities have a population under 5,000 inhabitants. In 2006, about 28% of individuals lived over 30 km (16% over 60 km) away from the hospital of reference and 10% of the population was over 65 years of age [17]. Healthcare services are organised according to 11 Health Areas and 248 Basic Health Zones. There are 14 public hospitals—at least one per Health Area—243 primary care centers, and 3,875 local primary care clinics [18]. Local primary care clinics service towns over 50 inhabitants without access to a primary care center. Smaller towns have house-calls available upon request [19].

Spanish pre-hospital emergency services have been described in detail elsewhere [11]. Regular primary care visits are managed by primary care centers and local primary care clinics. Morning-only opening hours are available in rural areas while both morning and afternoon opening hours are available in urban areas. The 220 PCEC are located in healthcare centers and, in exceptional cases due to high population dispersion, in local primary care clinics. PCEC provide out-of-hours emergency primary care services. Each municipality has an assigned hospital, called the reference hospital, and a PCEC where emergency care is provided to cases not requiring diagnostic tests such as X-rays or laboratory tests.

### Data sources

Individual-level data were compiled from the 2003 Regional Health Survey of Castile and Leon, a representative survey of the region that targets the population aged 16 and over. The complete microdata file was extracted from the 2003 National Health Survey, provided by the Ministry of Health and Consumer Affairs. The effective sample for Castile and Leon was 97.9% of the theoretical sample. The sampling frame for the national survey consisted of all non-institutionalized persons residing in Spain. A detailed description of the survey methodology can be found in our study [9] and on the Spanish National Institute of Statistics website [20].

We merged the individual-level data with two sources of municipality-level data in order to include relevant contextual variables. Information on availability of PCEC in the municipality of residence was obtained from the Guidelines for Health Planning for Castile and Leon [18]. Municipality-level average socioeconomic status (SES) variables and the proportion of households without a vehicle were obtained from the 2001 Population and Housing Census [21].

### Definition of variables

The dependent variable of interest was PCEC utilization in the last 12 months. Respondents were asked “*In the last 12 months, have you used any emergency service for a problem or disease?”* Those responding affirmatively were asked *“With regard to the last time you used an emergency service in these 12 months, what type of service was it?”* Those responding that it was a national healthcare emergency care center or national healthcare care center other than a hospital, were considered to have used public primary health emergency services. These respondents were then asked: *“Where were you attended?”* Only those responding “in an emergency center” were considered to have used a public PCEC. In addition, persons who reported having been admitted to a hospital during the last 12 months were asked the reason for the most recent admission. Women who were last admitted to deliver a baby were excluded.

#### Individual-level variables

The sociodemographic variables included were sex, age and SES, defined by the occupation of the household’s primary breadwinner. Thus, individuals were categorized as high SES (managers/white-collar employees) or middle-low SES (blue-collar/manual workers). We used self-rated health in the last 12 months as a proxy for health status, dichotomized as “positive” for respondents who rated their health as “good” or “very good”, and “negative” for those who reported their health as “fair”, “poor”, or “very poor”. The presence of chronic diseases was determined by asking respondents if a physician had diagnosed them with any condition from a list of 18 chronic diseases. Responses were grouped into 3 categories: no chronic disease, 1–2 chronic diseases, and more than 2 chronic diseases. Finally, we collected information regarding changes in regular activity. Respondents were asked whether or not they had reduced their regular activity in the last 2 weeks for at least half a day due to pain or any other symptoms (Yes/No).

#### Contextual variables

Municipalities were classified according to availability of emergency care centers into: municipalities with HES and with PCEC, municipalities with PCEC only, and municipalities without emergency care centers. The proportion of households without a vehicle, average municipal SES, percentage of persons ≥65, and the index of dependence (defined as the percentage of the population aged <16 and the population age ≥65 with respect to the population 16–64 years of age) were collected from census data as continuous variables and categorized as tertiles.

### Statistical analysis

First, we described the data at the univariate level using frequency analyses. We then conducted 2-level multivariate logistic regression analyses in order to quantify the independent effect of the availability of emergency care centers in the municipality of residence on PCEC utilization. Individuals (first level) were grouped by municipality of residence (second level).

#### Fixed-effects analysis

We examined the association between PCEC utilization and individual- and contextual-level variables. Odds ratios (OR) and their 95% confidence intervals (CI) were obtained from the beta coefficients (standard errors) in the fixed part of the model.

Bivariate collinearity among independent variables was evaluated by calculating Spearman’s rho (ρ) correlation coefficient. Variables with a ρ ≥0.60 were not analyzed together in the multivariate model.

#### Random-effects analysis

To explore whether PCEC utilization was more similar among individuals living in the same municipality than among those in different municipalities, we calculated the intraclass correlation coefficient (ICC), the proportion of variance explained (PVE) due to differences between municipalities, and the median odds ratio (MOR), as follows:

- *ICC* = (*V*_*m*_)/(*V*_*m*_ + *V*_*i*_) × 100, where: V_m_ = variance between municipalities and V_i_ = individual variance. Since the dependent variable is dichotomous, the ICC was calculated using the method of Snijders and Bosker, where V_i_ = π^2^/3 [22].
- *PVE* = (*V*_0_ - *V*_1_)/(*V*_0_) × 100, where V_0_ = second-level variance of the null model, and V_1_ = second-level variance of the adjusted model.
- *MOR* = *exp* [√ (2*xV*_*m*_) × 0.675] where V_m_ is the area level variance, and 0.6745 is the 75th centile of the cumulative distribution function of the normal distribution with mean 0 and variance 1 [23]. When randomly picking out two municipalities, MOR represents the median value of the odds ratio between the municipality at highest risk and the municipality at lowest risk. In this study, the MOR shows the extent to which the individual probability of using PCEC is determined by the municipality. Weighted coefficients for the region of Castile and Leon were used in all the analyses. The parameters were estimated by maximum likelihood with adaptive quadrature using the GLLAMM program [24]. The Stata software package, version 11.00, was used to perform the analyses [25].

### Qualitative phase of the study

#### Geographical context

The qualitative phase of the study was conducted in Segovia, which was chosen because it is the province within the Castile and Leon region with one of the greatest population dispersion. The population of Segovia (164,268 inhabitants), is distributed across 217 municipalities according to the Spanish National Institute of Statistics [16]. This province is divided into 16 Basic Health Zones, 3 of which are located in urban areas and 13 in rural areas. Each Basic Health Zone includes at least one PCEC [18].

### Qualitative design

The field work was conducted between January 2008 and December 2009, when the qualitative data collection period ended according to the project schedule. We designed a qualitative phase linked to the aim of this study that included 14 in-depth interviews of 6 general practitioners and 8 primary care nurses, by snowball sampling. These healthcare professionals worked in three of the PCEC available across the thirteen rural Basic Health Zones in the province of Segovia. These PCEC are located in the biggest municipality of the Basic Health Zone, and have to give healthcare at all the little municipalities (villages) that make up each zone. Distances between PCEC and the reference hospital ranged from 30 to 95 kilometers. We used a scripted interview specifically designed to collect the healthcare professionals’ perceptions regarding the use of rural PCEC and the factors determining such use patterns. One of the researchers involved in the study, recrutined the respondents and conducted the interviews. None of the healthcare professionals who were invited to be interviewed refused to participate. The interviews were conducted in Spanish, the mother tongue of the interviewer and interviewees, and recorded with the interviewee’s informed verbal consent. Interview transcriptions in Spanish were entered into Open Code 3.4 for content analysis.

### Qualitative analysis

In this phase of the study all transcripts were analysed using qualitative content analysis, focusing on aspects related to health services utilization, PCEC utilization specifically [26]. All the interview transcripts were read several times to allow for note-taking [27]. From these readings and notes, meaning units referring to PCEC utilization were identified by one of the researchers, which led to corresponding codes to label such units. Codes were then grouped to form the condensed meaning units by two of the researchers. Finally, one sub-theme emerged from each condensed meaning unit with PCEC utilization as the only theme under study.

### Role of researchers

The researchers did not occupy dual roles of like clinician and researcher.

### Ethical considerations

The research protocols, including quantitative and qualitative phase*,* were approved by the ethics committee of the Health Institute Carlos III.

## Results

### Quantitative phase of the study

Once we excluded admissions of women giving birth (n = 23) and missing values (n = 13), the Regional Health Survey of Castile and Leon included 4,282 records (weighted data) of residents from 179 municipalities. In the last 12 months, 21.2% of the population reported using some type of public emergency care service versus 1.4% that reported using a private emergency care service. Regarding the last public emergency care service used in the last 12 months, 6.7% of the population had used pre-hospital emergency services (6.2% PCEC), and 14.4% of the population used public HES. In addition, 0.3% of the population received care for the last emergency in the last 12 months where the patient was located at the moment of the emergency (residence, place of work) or at a mobile unit.

Table 1 shows the frequency distribution of the variables under study according to the availability of emergency care centers in the municipality of residence. Areas with no emergency care centers tend to have an older population, a higher proportion of individuals suffering from chronic diseases, a higher percentage of households without vehicles, and a lower SES than municipalities with emergency care centers. The highest proportion of PCEC utilization (11.2%) is recorded in those municipalities with PCEC but no HES.

**Table 1:** **Description of the sample**

	Municipal healthcare resources							
VARIABLES	Hospital + PCEC		PCEC only		Neither hospital nor PCEC		Total	
Individuals variables	n	%	n	%	n	%	n	%
Sex								
- Female	1102	(52.73)	466	(49.00)	593	(47.78)	2161	(50.47)
- Male	988	(47.27)	485	(51.00)	647	(52.14)	2120	(49.51)
Age								
- 15–34 years old	684	(32.73)	274	(28.81)	310	(24.98)	1268	(29.61)
- 35–64 years old	997	(47.70)	423	(44.48)	506	(40.77)	1926	(44.98)
- 65 years old and older	409	(19.57)	255	(26.81)	424	(34.17)	1088	(25.41)
Reduction of regular activity								
- No	1881	(90.00)	860	(90.43)	1127	(90.81)	3868	(90.33)
- Yes	209	(10.00)	91	(9.57)	113	(9.11)	413	(9.65)
Self-rated health								
- Negative	543	(25.98)	281	(29.55)	420	(33.84)	1244	(29.05)
- Positive	1547	(74.02)	671	(70.56)	820	(66.08)	3038	(70.95)
Number of chronic conditions								
- None	1071	(51.24)	478	(50.26)	562	(45.29)	2111	(49.30)
- 1-2 conditions	724	(34.64)	351	(36.91)	465	(37.47)	1540	(35.96)
- Over 2 conditions	295	(14.11)	122	(12.83)	213	(17.16)	630	(14.71)
SES								
- IV-V (low)	1050	(50.24)	581	(61.09)	604	(48.67)	2235	(52.20)
- I-III (high)	1040	(49.76)	371	(39.01)	637	(51.33)	2048	(47.83)
**Municipal-level variables**								
Average SES level, tertiles								
- T1 (lowest)	138	(6.60)	500	(52.58)	730	(58.82)	1368	(31.95)
- T2	1130	(54.07)	217	(22.82)	198	(15.95)	1545	(36.08)
- T3 (highest)	822	(39.33)	234	(24.61)	312	(25.14)	1368	(31.95)
% Households without a vehicle, tertiles								
- T1 (up to 30%)	918	(43.92)	263	(27.66)	308	(24.82)	1489	(34.77)
- T2 (30-35%)	836	(40.00)	319	(33.54)	212	(17.08)	1367	(31.92)
- T3 (over 35%)	336	(16.08)	369	(38.80)	721	(58.10)	1426	(33.30)
PCEC utilization								
- No	2001	(95.74)	845	(88.85)	1173	(94.52)	4019	(93.86)
- Yes	89	(4.26)	107	(11.25)	68	(5.48)	264	(6.17)
TOTAL	2090	(48.81)	951	(22.21)	1241	(28.98)	4282	(100.00)

Regional Health Survey of Castile and Leon, 2003.

PCEC: Primary care emergency center; n: frecuency; SES: Socio-economic Status; T: tertiles.

Table 2 shows PCEC utilization by different population groups. Percentages are higher among men than women (6.8% vs. 5.5%), among younger than older persons (8.8% among 15–34 year-olds vs. 5% among those older than 34 years of age), among those reporting poorer health status (7.6% vs. 5.6%), and among individuals with low SES (7.5% vs. 4.6%). At the municipality level, people living in municipalities with lower average SES or in municipalities with a PCEC but no HES also report a higher percentage of PCEC utilization (PCEC only: 11.2% vs. PCEC + HES: 4.3%).

**Table 2:** **Utilization of PCEC**

	***In the last 12 months, have you used a PCEC for a problem or disease?***					
VARIABLES	Yes		No		Total	
Individual variables	n	%	N	%	n	%
Sex						
- Female	118	(5.46)	2043	(94.54)	2161	(50.46)
- Male	145	(6.84)	1976	(93.16)	2121	(49.52)
Age						
- 15–34 years old	112	(8.83)	1156	(91.17)	1268	(29.61)
- 35–64 years old	101	(5.24)	1825	(94.76)	1926	(44.97)
- 65 years old. and over	50	(4.60)	1038	(95.40)	1088	(25.40)
Reduction of regular activity						
- No	223	(5.77)	3645	(94.23)	3868	(90.31)
- Yes	40	(9.66)	374	(90.34)	414	(9.67)
Self-rated health						
- Negative	94	(7.56)	1150	(92.44)	1244	(29.05)
- Positive	169	(5.56)	2868	(94.44)	3037	(70.91)
Number of chronic conditions						
None	119	(5.64)	1992	(94.36)	2111	(49.29)
1-2 conditions	100	(6.49)	1441	(93.51)	1541	(35.98)
Over 2 conditions	44	(6.98)	586	(93.02)	630	(14.71)
SES						
- IV-V (low)	168	(7.52)	2066	(92.48)	2234	(52.16)
- I-III (high)	95	(4.64)	1953	(95.36)	2048	(47.82)
**Municipal-level variables**						
Emergency care centers						
- HES and PCEC	89	(4.26)	2001	(95.74)	2090	(48.80)
- PCEC only	107	(11.24)	845	(88.76)	952	(22.23)
- None	68	(5.48)	1173	(94.52)	1241	(28.98)
Average SES, tertiles						
- T1 (lowest)	110	(8.04)	1259	(91.96)	1369	(31.96)
- T2	92	(5.95)	1454	(94.05)	1546	(36.10)
- T3 (highest)	62	(4.53)	1307	(95.47)	1369	(31.96)
% Households without a vehicle, tertiles						
- T1 (up to 30%)	74	(4.97)	1414	(95.03)	1488	(34.74)
- T2 (30-35%)	105	(7.68)	1262	(92.32)	1367	(31.92)
- T3 (over 35%)	83	(5.82)	1343	(94.18)	1426	(33.29)
TOTAL	262	(6.12)	4019	(93.86)	4281	(100.00)

Regional Health Survey of Castile and Leon, 2003.

PCEC: Primary care emergency center; n: frecuency; SES: Socio-economic Status; HES: Hospital Emergency Services; T: tertiles.

Table 3 shows the fixed-effects corresponding to both the individual- and contextual-level variables included in the multivariate model. Individuals residing in a municipality with PCEC as the only emergency care service available are 2.46 times as likely to use it as those living in a municipality with PCEC and HES. Further, PCEC utilization among residents of municipalities with no emergency care centers did not differ from PCEC utilization among those in municipalities with both PCEC and HES. Other variables such as sex, age, health status, and SES were also significantly associated with PCEC utilization as described below.

**Table 3:** **Estimates for fixed effects between individual and contextual-level characteristics on PCEC utilization**

VARIABLES		MODEL 1		MODEL 2			MODEL 3			MODEL 4		
Individual variables	p	OR	95% CI	p	OR	95% CI	p	OR	95% CI	p	OR	95% CI
Sex *(ref: female)*												
- Male	0.094	1.25	(0.96;1.62)	0.031	1.32	(1.03;1.71)	0.029	1.33	(1.03;1.72)	0.028	1.33	(1.03;1.73)
Age *(ref: 15–34 years old.)*												
- 35–64 years old	0.001	0.54	(0.38;0.77)	<0.001	0.46	(0.32;0.67)	<0.001	0.46	(0.32;0.67)	<0.001	0.46	(0.31;0.66)
- 65 years old and over	<0.001	0.44	(0.28;0.67)	<0.001	0.28	(0.17;0.46)	<0.001	0.28	(0.17;0.46)	<0.001	0.28	(0.17;0.46)
Reduction of regular activity *(ref: no)*												
- Yes	0.014	1.84	(1.13;2.99)	0.108	1.55	(0.91;2.65)	0.111	1.55	(0.90;2.65)	0.104	1.56	(0.91;2.67)
Self-rated health *(ref: negative)*												
- Positive	0.011	0.70	(0.53;0.92)	0.003	0.62	(0.45;0.85)	0.004	0.62	(0.45;0.86)	0.003	0.62	(0.45;0.85)
Number of chronic conditions *(ref: none)*												
- 1-2 conditions	0.462	1.14	(0.81;1.59)	0.127	1.31	(0.93;1.84)	0.123	1.31	(0.93;1.84)	0.117	1.31	(0.93;1.84)
- Over 2 conditions	0.29	1.19	(0.86;1.65)	0.028	1.56	(1.05;2.31)	0.017	1.61	(1.09;2.39)	0.018	1.61	(1.08;2.38)
SES (*ref: low -IV, V*)												
- I-III (high)	0.005	0.64	(0.47;0.87)	0.014	0.68	(0.50;0.92)	0.02	0.70	(0.52;0.94)	0.022	0.70	(0.52;0.95)
Municipal-level variables												
Emergency care centers *(ref: HES + PCEC)*												
- PCEC only	0.001	2.45	(1.48;4.05)				<0.001	2.57	(1.56;4.25)	<0.001	2.46	(1.49;4.05)
- No emergency care	0.863	1.05	(0.63;1.73)				0.579	1.16	(0.69;1.93)	0.528	1.18	(0.70;1.99)
Average SES (*ref: T1- lowest*)												
- T2	0.976	1.01	(0.59;1.73)							0.854	1.05	(0.63;1.76)
- T3 (highest)	0.264	0.74	(0.43;1.26)							0.272	0.75	(0.44;1.26)
% Households without a vehicle, *(ref:T1: up to 30%)*												
- T2 (30-35%)	0.115	1.62	(0.89;2.96)							0.186	1.45	(0.84;2.49)
- T3 (over 35%)	0.955	0.98	(0.58;1.68)							0.977	0.99	(0.59;1.67)

PCEC: Primary care emergency center; p: probability; OR: odds ratio; CI: confidence intervals; SES: Socio-economic Status; HES: Hospital Emergency Services; T: tertile.

Model 1: univariate estimates; Model 2: Individuals variables; Model 3: Model 2 + emergency care centers; Model 4:individual variables + contextual variables.

Table 4 provides summary results from random-effects multilevel models that examined the relative contribution of individual- and contextual-level variables of the municipality of residence to the individual’s utilization of a PCEC. The null model illustrates the high variability in PCEC use across municipalities (variance = 1.064; standard error = 0.219). Of the total variance, 23.9% is explained by differences in municipalities (intraclass correlation (ICC) = 23.9). The availability of emergency care centers in the municipality explained an additional 23.5% of the variance in PCEC use across municipalities. Including both individual- and contextual-level variables explained 31.2% of the level 2 variance. The person’s median probability of using PCEC is 2.7 times higher if this persons moved to a municipality with higher utilization (MOR = 2.7). This median probability decreases to 2.2 in Model 4.

**Table 4:** **“Random-effects” multilevel models**

MULTILEVEL MODELS	Municipality-level variance (SE)		PVE (%)	MOR	ICC
*Null model*	1.064	(0.219)	reference	2.67	23.9
*Univariate models:*					
Sex	1.059	(0.219)	0.49	2.67	23.8
Age	1.090	(0.230)	-2.49	2.71	24.3
Reduction of regular activity	1.079	(0.225)	-1.43	2.69	24.1
Self-rated health: positive	1.092	(0.226)	-2.66	2.71	24.4
Number of chronic conditions	1.062	(0.218)	0.22	2.67	23.8
SES	1.015	(0.215)	4.60	2.61	23.0
Municipal emergency care centers	0.814	(0.219)	23.53	2.36	19.4
% of households without a vehicle	0.991	(0.208)	6.84	2.58	22.6
Average SES level of municipality	1.019	(0.209)	4.22	2.62	23.1
*Multivariate models*					
Model 2: Invididual-level variables	1.073	(0.237)	-0.88	2.68	24.0
Model 3: Model 2 + emergency care centers	0.819	(0.241)	23.01	2.37	19.5
Model 4: Individual- + contextual-level variables	0.725	(0.230)	31.82	2.25	17.6

Relative contribution of individual- and contextual-level characteristics of living in a municipality, to the individual’s utilization of a PCEC.

PCEC: Primary emergency care center; SE: Standard Error; PVE: percentage of variance explained; MOR: median odds ratio; ICC: Intraclass correlation; SES: Socio-economic Status.

### Qualitative phase of the study

The theme “PCEC utilization” consisted of three subcategories which emerged during the axial coding phase: a) Distance affects PCEC utilization; b) PCEC utilization as local primary care clinics; c) Higher PCEC utilization by students, workers, and chronic disease patients (Table 5).

**Table 5:** **Theme, sub-theme and codes**

Theme	Sub-theme	Condensed meaning unit	Codes	Meaning unit
PCEC Utilization	Distance affects PCEC use	-Greater PCEC utilization in rural than in urban areas.	-Access	-PCEC use according to distance-accessibility
		-Greater PCEC utilization in the same towns where located.	-City/Urban area	
		-Lower PCEC utilization due to distance.	-Distance	
			-Hospital Emergency Services	
			-Isolated villages	
			-No public transport	
			-Lack of private transportation	
			-PCEC use	
	Utilization of PCEC as regular local primary care office	-Greater PCEC utilization on weekends.	-High utilization on weekends.	-Description of PCEC use
		-PCEC used as afternoon-hours local primary care office.	-Access barrier.	
			-Morning hours only doctors’ office	
			-Use as regular doctor’s visit.	
	Greater PCEC utilization by working and student population and by chronic disease patients	-PCEC use by working adults.	-Students	-PCEC clients profile
		-PCEC use by students.	-Working adults	
		-PCEC use by chronic patients.	-Chronic patients	
			-PCEC use	

PCEC: Primary emergency care center.

#### Distance affects PCEC utilization

One of the perceptions of primary healthcare professionals is that PCEC use is influenced, first of all, by the distance between the municipality where the demand arises and the reference hospital, located in the urban center. When said distance to the HES is greater than the distance to the PCEC, patients choose the latter as their first option. Interviewees also feel that individuals residing in villages use PCEC more often than city-dwellers.

*“I think that in a capital city (urban center) these (*PCEC*) are not used as much, people go to the hospital directly, but in isolated villages they do use them. Many people go, for whatever reason, from chest pains to the occasional heart attack, adolescents getting drunk at the town summer festivals, car accidents, skin cuts at the pool,…it’s the ‘catch-all’.*

Additionally, healthcare professionals feel that distance acts as a barrier to healthcare access for those individuals residing in a village with no PCEC and who do not have private means of transportation to get to one. This is also due to the lack of public transportation between these towns and those with PCEC.

*“There is another important sector in demand for PCEC: the elderly or many mothers with children coming from tiny villages with no public transportation, and who wait for the husband to return from work, or for their son, or whoever with a car to take them”*

#### PCEC utilization as local primary care clinics

Healthcare professionals describe how PCEC is sometimes used as a “regular visit to the doctor,” in most cases as “out-of-hours primary care office,” instead of as PCEC.

*“A substantial proportion of PCEC visits is the same as that in a regular doctor’s office. Those patients that either couldn’t make it to their regular doctor’s morning hours or those who know that going to a primary care emergency center is faster than a regular visit to their doctor come to the* PCEC*”.*

The main reason identified by the healthcare professionals for the utilization of PCEC as “out-of-hours” or “afternoon doctor’s office” in this rural context is the scheduling incompatibility between local primary care office hours and work and educational obligations that usually take place outside the municipality of residence.

*“A substantial number of emergencies are related to people who can’t easily get permission to take time off from work to go to the doctor in their town because many of them don’t work in their town but in Segovia or other urban centers and thus, going to the doctor’s means not returning to work [that day]. So, some people don’t ask for time off because, as they say, “By the third time I ask my boss for time off, he’ll fire me.”*

*“More people go to the PCEC on weekends because, for example, their kids who are studying elsewhere come home and they all go to the town on weekends with their parents […]”*

#### Higher PCEC utilization by students, workers, and chronic disease patients

PCEC’s more convenient opening hours for workers and students when compared to the morning-only schedule of regular local primary care clinics in rural areas influences the profile of PCEC users profile: mostly men and youth.

*“You don’t see young people at the regular doctor’s office in the mornings but later, in the afternoon, after work, at the* PCEC*, they come to primary care emergency and they are seen then”.*

Healthcare professionals see another sub-population, chronic patients, as another group that poses greater demand on PCEC than other populations. This utilization is considered a sign of lack of the patient’s empowerment over the control of their disease.

*“Chronic ones (patients) also come. Anything that worries the chronic patient, so, just as easy as they would go to the doctor’s office regular morning hours, they come in the afternoons and even at night to the* PCEC*, just as easy, […] these visits are, in my opinion, due to a lack of information regarding their chronic condition”.*

## Discussion

### Main findings

Individuals residing in a municipality with only PCEC as emergency care center, were 2.46 times as likely to use this service as those living in a municipality with HES and PCEC. Further, there were no statistically significant differences in the probability of using PCEC between respondents residing in municipalities with no emergency care centers and those in municipalities with both HES and PCEC. Sex, age, health status, and SES were also significantly associated with PCEC use. At the contextual level, availability of emergency care centers in the municipality explained 23.1% of the variability in PCEC utilization among municipalities.

From the qualitative phase of the study, we found that healthcare professionals in rural areas believe that as the distance from the patient’s residence to the hospital increases so does PCEC utilization. They alsoconsider that the distance from residence to PCEC is an access barrier for individuals without their own means of transportation. Finally, the healthcare professionals explained the overrepresentation of workers and students among PCEC users by the conflict between those patients’ schedules and the morning-only opening hours of the local primary care office in rural areas.

### Possible explanations

Regarding the last emergency care service used in the last 12 months, the percentage of PCEC utilization (6.2%) in the Castile and Leon region is comparable to the national average for the same year (6.3%). However, though these data show that PCEC utilization in the region of Castile and Leon is comparable to the national average, they do not provide *actual* utilization rates since the survey question only asks about the last emergency care service used by the respondent.

Our results do lead to two important findings related to the fixed effects and emergency care centers locations. First, PCEC utilization is greater among those individuals residing in municipalities with PCEC as the only emergency care service in comparison to those living in municipalities with both PCEC and HES available. Previous research on European care delivery systems shows that, despite the fact that very different models of management of emergency care services (e.g., deputizing services, emergency departments, primary care centers, practice-based services, and telephone assistance and triage) co-exist, distance to healthcare centers negatively impacts their utilization. This decrease in utilization as distance increases affects both face-to-face service (primary care centre or home visit) as well as the rates of telephone calls to emergency care services [28–31]. In this regard, we benefited from the methodology applied in this article—a mixed-methods approach—which facilitated drawing on the insights of healthcare professionals working in rural areas regarding how distance to HES increases PCEC use.

Further, the second major finding is that the probability of PCEC utilization by individuals residing in municipalities with no emergency care centers is similar to that of residents of municipalities with both PCEC and HES. Based on a recent study by the authors showing that as distance from the municipality to the hospital increases, the probability of the munipality’s residents using HES decreases [9], one could argue that the populations residing in municipalities with no emergency care center encounter similar access barriers to both PCEC and HES.

In this study, the mixed-methods approach allows further clarification by having healthcare professionals identify distance to PCEC as an access barrier to the center and, further, that distance acts as more of a barrier to individuals who depend on third parties for car rides and/or public transportation to get to the center. It is possible that in regions with high population dispersion, lack of access to HES may increase PCEC utilization, but only among residents of municipalities with a PCEC. In this work, the location of the emergency care centers is the independent variable with the strongest relationship to PCEC utilization, explaining close to a quarter of the variability of PCEC use across municipalities. This is a key issue regarding the goal of equal access which requires further study.

However, the greater PCEC utilization by men than women observed in both, the survey data and healthcare professionals’ opinions, challenges current scientific evidence. Results from several research studies consistently show women as the more frequent users of primary care emergency services [32–34]. Healthcare professionals identified the incompatibility between the usual work schedule in rural areas and regular opening hours of the primary care office (mornings only) as an important access barrier which would lead to overrepresentation of males among rural areas emergency care patients. Furthermore, the higher rates of formal employment among men than women in this region would explain the greater impact of the schedule conflict on men [35].

The significant and independent effect of age revealed that as age increases, the likelihood of using a PCEC decreases. Research using Spanish national data as well as other research comparing European countries reveals that working adults are the most likely users of out-of-hours primary care [32, 34, 36]. Supporting and adding some depth to these findings, healthcare professionals interviewed here reported a greater PCEC use by students and workers which, again, they associated with the difficulty of attending a primary care facility in rural areas during regular opening hours. Lower utilization rates by older age groups have been reported in Spain previously [36], although the majority of European countries observe an increase in PCEC among older age groups [34]. The lower attendance to PCEC of older individuals that we found may reflect access and/or utilization barriers beyond the ones included in our model [37] such as a greater ease of access to local primary care clinics given this group’s greater availability of free time in the mornings. In fact, recent research by Borda and colleagues [38] in this same population and region, reported that the rates of avoidable hospitalization declined with widening distance from municipalities to the hospital and increasing proportion of older people in the municipality, suggesting that primary care center in rural areas offer adequate and accessible care.

### Study limitations

These findings should be interpreted in the context of the study’s limitations. First, data collection of qualitative and quantitative study was conducted at different times. The Regional Health Survey Castile and León was compiled in 2003, and the qualitative fieldwork was conducted in 2009. During this time period, health resources are no change in the area. On the other hand, the only significant socio-demographic change in the region is increasing migrant population. However, this increase was similar in rural and urban areas, so it is not possible that this fact biases our results. Second, the information collected in the survey was self-reported, which could result in an underestimation of PCEC use. Third, the sampling framework consisted of non-institutionalized persons only which excludes a certain proportion of older persons who live in senior care residences or assisted-living facilities and who, presumably, use public PCEC. Due to multicollinearity with the dependent variable, the contextual variables index of dependence and the percentage of persons aged 65 or over in the municipality could not be included in the model. Finally, we had no individual-level information on variables particularly relevant based on our qualitative findings such as the availability of private transportation in the respondent’s home.

Further research is needed to gain a deeper understanding of the current attitudes and practices of the rural population, both men and women regarding utilization of primary care emergency to better match the supply and demand.

## Conclusions

In summary, we conclude that the location of healthcare centers is substantially associated with their utilization. Further research is required to determine whether lower PCEC use among the subpopulations identified here reflects disparities in access to healthcare centers. Additionally, tailoring local primary care office opening hours (i.e., extending office hours) to the needs of the working population in rural areas may: 1) translate into much more effective and timely delivery of emergency care by PCEC staff, by reducing the number of non-emergency cases; and 2) result in higher rates of uninterrupted patient follow-up by facilitating all regular care to take place at local primary care clinics.

## Abbreviations

PCEC: Primary care emergency centers
SES: Socioeconomic status
OR: Odds ratio
95% CI: 95% confidence interval
ρ: Spearman’s rho correlation coefficient
ICC: Intraclass correlation coefficient
PVE: Proportion of variance explained
V_m_: Variance between municipalities
V_i_: Individual variance
V_1_: Second-level variance of the adjusted model
V_0_: Second-level variance of the null model
MOR: Median odds ratio.
